# *Rice black‐streaked dwarf virus*‐encoded P5‐1 regulates the ubiquitination activity of SCF E3 ligases and inhibits jasmonate signaling to benefit its infection in rice

**DOI:** 10.1111/nph.16066

**Published:** 2019-08-09

**Authors:** Long He, Xuan Chen, Jin Yang, Tianye Zhang, Juan Li, Songbai Zhang, Kaili Zhong, Hengmu Zhang, Jianping Chen, Jian Yang

**Affiliations:** ^1^ State Key Laboratory for Quality and Safety of Agro‐products Institute of Plant Virology Ningbo University Ningbo 315000 China; ^2^ State Key Laboratory Breeding Base for Zhejiang Sustainable Pest and Disease Control, Zhejiang Provincial Key Laboratory of Plant Virology Institute of Virology and Biotechnology, Zhejiang Academy of Agricultural Sciences Hangzhou 310021 China; ^3^ College of Plant Protection Nanjing Agricultural University Nanjing 21000 China; ^4^ College of Plant Protection Hunan Agricultural University Changsha 410000 China; ^5^ College of Forestry and Biotechnology Zhejiang A&F University Linan 311300 China; ^6^ College of Agriculture and Biotechnology Zhejiang University Hangzhou 310058 China; ^7^ Institute of Plant Protection Hunan Academy of Agricultural Sciences Changsha 410000 China

**Keywords:** jasmonic acid pathway, rice, *Rice black streaked dwarf virus* (RBSDV), SCF complex, ubiquitination

## Abstract

- SCF (Skp1/Cullin1/F‐box) complexes are key regulators of many cellular processes. Viruses encode specific factors to interfere with or hijack these complexes and ensure their infection in plants. The molecular mechanisms controlling this interference/hijack are currently largely unknown.
- Here, we present evidence of a novel strategy used by *Rice black‐streaked dwarf virus* (RBSDV) to regulate ubiquitination in rice (*Oryza sativa*) by interfering in the activity of OsCSN5A. We also show that RBSDV P5‐1 specifically affects CSN‐mediated deRUBylation of OsCUL1, compromising the integrity of the SCF^COI1^ complex.
- We demonstrate that the expressions of jasmonate (JA) biosynthesis‐associated genes are not inhibited, whereas the expressions of JA‐responsive genes are down‐regulated in transgenic P5‐1 plants. More importantly, application of JA to P5‐1 transgenic plants did not reduce their susceptibility to RBSDV infection.
- Our results suggest that P5‐1 inhibits the ubiquitination activity of SCF E3 ligases through an interaction with OsCSN5A, and hinders the RUBylation/deRUBylation of CUL1, leading to an inhibition of the JA response pathway and an enhancement of RBSDV infection in rice.

## Abstract 2

See also the Commentary on this article by https://doi.org/10.1111/nph.16204.

## Introduction

Plants are constantly under pressure from various biotic and abiotic stresses. To counteract these stresses, plants have evolved their unique proteomic plasticity, which is partially driven by the ubiquitin proteasome system (UPS). The UPS is a highly regulated protein modification process that controls most protein degradation events and is highly conserved among eukaryotes (Khoury *et al*., 2011; Zhou & Zeng, 2017). Ubiquitination is an enzymatic process through which ubiquitin (Ub) moieties are covalently attached to protein substrates for intracellular degradation. This process is mediated by an enzymatic cascade involving an E1 Ub‐activating enzyme, an E2 Ub‐conjugating enzyme, and an E3 Ub‐ligase to transfer Ub molecules from the E2 enzyme to protein substrates. The E3 ligases are classified into two groups according to their structural features: single components and multi subunit complexes. The E3 single component ligases consist of the homologous to the E3‐associated protein C‐Terminus (HECT), Really Interesting New Gene (RING) and U‐Box subgroups, and the most common E3 multi subunit complexes are the Cullin‐RING Ligases (CRLs) (Mazzucotelli *et al*., 2006). In plants, CRLs are the most abundant E3 ligases and exist as complexes with a cullin subunit. Three main types of cullin (CUL1, CUL3 and CUL4) have been reported in various plants (Hotton & Callis, 2008). Among these cullins, the Cullin1‐based group, also known as the Skp1/Cullin1/F‐box (SCF) complex, is the largest group, and their substrates are recognized by the F‐box protein (Hua & Vierstra, 2011). The SCF complex is currently the best characterized complex, mainly due to its irreplaceable roles in cellular processes such as organ identity, floral development, light signaling and hormonal responses (Wang *et al*., 2003; Marrocco *et al*., 2006; Santner & Estelle, 2009; Cheng *et al*., 2011).

The activities of CRLs are regulated by various mechanisms, including cycles of covalent attachment and removal of a ubiquitin‐like protein known as ‘Related to Ubiquitin’ (RUB) in plant cells and ‘Nedd8’ in animal and yeast cells (Schwechheimer, 2018). The COP9 signalosome (CSN) complex is the most important regulator of these cycles (Wei *et al*., 2008). The CSN complex was reported to have eight subunits (CSN1 to CSN8); the CSN5 subunit is currently the best characterized of these, and its isopeptidase activity controls the removal of the RUB moiety from the cullin component associated with CRLs (Wang *et al*., 2010; Jin *et al*., 2014). Ubiquitination‐mediated protein degradation has been shown to play crucial roles in stress response processes in plants (Trujillo & Shirasu, 2010; Zhou *et al*., 2014; Zhou & Zeng, 2017). For example, some reports have shown that plant viruses exploit the functions of SCF complexes for their infection in plants via various mechanisms. Thiel *et al*. (2012) showed that the *Beet necrotic yellow vein virus* (BNYVV)‐encoded P25 protein targeted the F‐box protein to reduce cell death while promoting rhizomania symptoms. *Polerovirus*‐encoded P0 protein has been reported to act as an F‐box protein for the modulation of gene silencing in cells through interaction with SCF complexes, leading to the degradation of AGO1 protein (Pazhouhandeh *et al*., 2006). *Nicotiana tabacum* Avr9/Cf‐9–INDUCED F‐BOX1 (ACIF1) has been reported to regulate the *Tobacco mosaic virus* (TMV)‐induced hypersensitive response (HR; Van den Burg *et al*., 2008).

It is well known that the ubiquitination pathway plays an important role in plant hormonal responses, and plant hormones, especially jasmonic acid (JA), play vital roles in plant immune responses (Santner & Estelle, 2009). The JA‐mediated plant defense response requires the SCF^COI1^ complex‐dependent degradation of JAZs. To counteract host defense, plant pathogens, including viruses, have evolved new strategies to interfere with the SCF^COI1^ complex‐dependent ubiquitination pathway. The C2 proteins of *Tomato yellow leaf curl virus* (TYLCV) and *Beet curly top virus* (BCTV) were found to interact with CSN5 to hinder the functions of plant SCF complexes and to alter the jasmonate defense signaling machinery (Lozano‐Duran *et al*., 2011). Many recent studies have focused on JA signaling in order to establish the mechanisms controlling the interactions between plants and viruses (Liu *et al*., 2004; Yang *et al*., 2008; He *et al*., 2017).

*Rice black‐streaked dwarf virus* (RBSDV) is a member of the genus *Fijivirus* in the family *Reoviridae*. RBSDV infection often results in severe losses in the production of rice (*Oryza sativa*), maize and other cereal crops (Bai *et al*., 2001; Zhang *et al*., 2001). RBSDV is transmitted by the small brown planthopper (SBPH, *Laodelphax striatellus*) in paddy fields. Infected rice plants often show severe leaf darkening and plant stunting (Zhang *et al*., 2001). RBSDV contains 10 double‐stranded RNA (dsRNA) segments (S1 to S10), and the size of RBSDV dsRNA segments ranges from *c*. 1.4 to 4.5 kilobases. Most of the RBSDV dsRNA segments encode single proteins, except for the S5, S7 and S9 segments, which encode two proteins each (Fang *et al*., 2001; Zhang *et al*., 2001). The functions of some of the RBSDV proteins have been identified. For example, RBSDV P8 and P10 proteins are the viral core capsid protein and the outer capsid protein, respectively (Liu *et al*., 2007a,b; Sun *et al*., 2013b). The RBSDV S7‐1 open reading frame (ORF) encodes a tubular protein, which is important for RBSDV virion movement between cells, and the RBSDV S7‐2 ORF encodes a potential F‐box protein, known as P7‐2 protein. The P7‐2 protein is likely to be incorporated into maize cellular SCF complexes through an interaction with the ZmSKP1 protein (Sun *et al*., 2013c; Wang *et al*., 2013). The RBSDV S5 segment is a bicistronic RNA and encodes two proteins (P5‐1 and P5‐2; Yang *et al*., 2014). It has been reported that the P5‐1, P6 and P9‐1 proteins form viroplasm inclusions, in which viral replication and assembly occurs (Wang *et al*., 2010; Akita *et al*., 2012; Sun *et al*., 2013a). At present, the role of P5‐1 in RBSDV infection in plants remains unknown.

In this study, we have shown that RBSDV P5‐1 is capable of facilitating RBSDV infection in rice, and further analyses found that this viral protein specifically interacts with OsCSN5A in cells. Our results demonstrated that knockdown of *OsCSN5A* in rice greatly enhanced rice susceptibility to RBSDV infection; by contrast, increased expression of *OsCSN5A* in rice through stable transformation enhanced rice resistance to RBSDV infection. We also found that CSN‐mediated deRUBylation of CUL1 is specifically inhibited by P5‐1, leading to the alteration of SCF‐mediated E3 ubiquitination, and that knockdown of *OsCUL1* in rice significantly enhanced rice susceptibility to RBSDV infection. Furthermore, we showed that the expressions of JA responsive genes are down‐regulated in transgenic P5‐1 plants. More importantly, when JA was applied to the P5‐1 transgenic plants, the RBSDV disease incidence was not changed, relative to the mock‐treated control plants. In summary, we have shown for the first time that RBSDV is capable of interfering with the host ubiquitination pathway and jasmonate signaling to enhance infection, as well as disease symptom development, in rice.

## Materials and Methods

### Plasmid construction

Full length *OsCSN5A* and *RBSDV S5‐1* genes were PCR amplified using primers P5 and P6, and P7 and P8. The resulting PCR products were cloned individually behind a CaMV35S promoter in the pGWB509 vector using Gateway technology (Invitrogen) to generate p35S:OsCSN5A and p35S:P5‐1, respectively. Full length *OsCSN5A* and its reverse‐complement sequence were then PCR amplified using primers P9 and P10, and P11 and P12, ligated to form a hairpin, and inserted into a *BamH*I and *Sac*I enzyme pre‐digested pCAMBIA1301 vector to generate the pOsCSN5A:RNAi plasmid. The same method was then used to generate the pOsCUL1:RNAi plasmid using primers P13 and P14, and P15 and P16.

For yeast two‐hybrid assays, full length or partial RBSDV *S5‐1* gene sequences were PCR amplified using primers P17 and P21; P17 and P22; P17 and P23; P17 and P24; P17 and P25; P18 and P21; P19 and P22; and P20 and P24, cloned individually into the pGADT7 vector to produce pBD‐P5‐1, pBD‐P5‐1^1‐540^, pBD‐P5‐1^1‐392^, pBD‐P5‐1^1‐231^, pBD‐P5‐1^1‐106^, pBD‐P5‐1^479‐937^, pBD‐P5‐1^230‐540^ and pBD‐P5‐1^106‐231^, respectively. Similarly, partial sequences of *OsCSN5A* were PCR amplified using primers P26 and P31; P28 and P32; P29 and P33; P30 and P34; and P27 and P34 to produce pAD‐OsCSN5A^1‐58^, pAD‐OsCSN5A^59‐178^, pAD‐OsCSN5A^179‐322^, pAD‐OsCSN5A^323‐359^ and pAD‐OsCSN5A^59‐359^.

For Co‐IP assays, *OsCSN5A* was PCR amplified using primers P35 and P36, and cloned into the pGWB514 vector with a hemagglutinin (HA)‐tag to generate pGWB514‐OsCSN5A. *RBSDV P5‐1* was PCR amplified using primers P37 and P38, and cloned into the pGWB511 vector with a Flag tag to generate pGWB511‐P5‐1. *OsCUL1, OsCUL3A* and *OsCUL4* were PCR amplified using primers P41 and P42, P43 and P44, and P45 and P46, and individually cloned into the pGWB514 vector with a HA tag to generate plasmids pGWB514‐OsCUL1, pGWB514‐OsCUL3A and pGWB514‐OsCUL4.

For OsJAZ8 degradation assays, *OsJAZ8* was PCR amplified using primers P39 and P40, and cloned into the pGWB5C vector with a green fluorescent protein (GFP) tag to generate pG‐WB5C‐OsJAZ8.

The PCR products used for cloning were all generated using the KOD DNA polymerase (Toyobo, Kita‐ku, Osaka, Japan). The PCR primers used in this study are listed in the Supporting Information Table S1.

### Generation and characterization of transgenic rice

*Oryza sativa* L. ssp. *japonica* rice cv Wuyujing No.3 was transformed with plasmids p35S:P5‐1, p35S:OsCSN5A, pOsCSN5A:RNAi and pOsCUL1:RNAi using an *Agrobacterium tumefaciens*‐mediated transformation approach. The P5‐1 transgenic lines were characterized using reverse transcription polymerase chain reaction (RT‐PCR) and Western blotting. The OsCSN5A, OsCSN5A RNAi and OsCUL1 RNAi transgenic lines were confirmed by quantitative reverse transcription polymerase chain reaction (qRT‐PCR) using specific primers P61 and P62, and P63 and P64. The selected T2 generation transgenic lines were then used for plant growth and virus infection assays. The RT‐PCR primers used in this study are listed in the Table S1.

### Plant growth and virus inoculation

Wild‐type (WT) rice cv Wuyujing No. 3 and transgenic plants were grown inside glass beakers. Two‐wk‐old seedlings were transplanted into soil in plastic pots and grown at 26°C with a 16 h : 8 h, light : dark photoperiod. *Nicotiana benthamiana* plants were grown inside a glasshouse at 22°C with a 16 h : 8 h, light : dark photoperiod and used for transient gene expression assays.

RBSDV inoculation was performed using the small brown planthopper (SBPH, *Laodelphax striatellus*) as described in previous studies (Zhou *et al*., 2010; He *et al*., 2017). Briefly, first‐ or second‐instar SBPH nymphs were allowed to feed on RBSDV‐infected rice plants for 3 d and then on the WT rice plants for *c*. 10 d. RBSDV viruliferous SBPHs were transferred onto rice seedlings with one to two leaves (three nymphs per plant) and allowed to feed on the seedlings for 3 d. After elimination of the insects, the inoculated rice plants were grown inside a glasshouse for symptom observation. RBSDV infection in the inoculated plants was determined by RT‐PCR using RBSDV‐specific primers P1 and P2 (Table S1).

### Quantitative reverse transcription polymerase chain reaction (qRT‐PCR)

Total RNA was isolated from individual rice samples harvested at 60 d post‐RBSDV inoculation (dpi) using Trizol reagent (Invitrogen) and stored at −80°C until use. First strand cDNA was synthesized using a First Strand cDNA Synthesis Kit (Toyobo, Kita‐ku, Osaka, Japan) and 1 μg total RNA per 20 μl reaction. The qRT‐PCR was conducted on an ABI7900HT Sequence Detection System (Applied Biosystems, Foster City, CA, USA) using an AceQ qPCR SYBR Green Master Mix (Vazyme, Nanjing, Jiangsu, China). At least three biological replicates, with three technical replicates each, were used for each treatment. Relative expression levels of JA‐responsive and JA biosynthesis genes were analyzed using the 2^−ΔΔC(t)^ method as previously described (Livak & Schmittgen, 2001). In each reaction, an actin gene was used as the internal reference. Primers used in the qRT‐PCR reactions are listed in Table S1.

### Northern blot analysis

Northern blotting was performed as previously described (Yang *et al*., 2017). In brief, 3 μg total RNA from a sample was loaded into a well of a 1.5% formaldehyde containing agarose gel and separated through electrophoresis. The separated RNA was then transferred onto Hybond‐N+ membranes (Amersham Bioscience) and cross‐linked for 2 h at 80°C. RBSDV genomic RNA was detected using DIG‐labeled DNA probes specific to the 3′‐terminus of RBSDV genomic RNA or the 3′‐terminus of RBSDV S8 or S10 RNA. The probes were produced using a DIG High Prime DNA Labeling and Detection Starter Kit II (Roche) according to the manufacturer's instructions. The Hybond‐N+ membranes were pre‐hybridized for 2 h followed by an overnight hybridization with a specific probe at 42°C. The detection signal was visualized using the Amersham Imager 600 (GE Healthcare Bio‐Sciences, Pittsburgh, PA, USA). The primers used to make the DNA probes are listed in Table S1.

### Western blot analysis

Western blotting was conducted as described by Yang *et al*. (2016), with modifications. The harvested plant tissues were ground individually in liquid nitrogen and then homogenized in a protein extraction buffer (Sigma‐Aldrich) supplemented with protease inhibitor cocktail tablets (Roche; 1 tablet : 50 ml buffer). After 20 min centrifugation at 18 000 ***g*** at 4°C, supernatants were collected from each sample and boiled for 5 min; the proteins were then separated in SDS‐PAGE gels through electrophoresis before being transferred to nitrocellulose membranes. Antibodies specific to RBSDV P8, RBSDV P10, OsCSN5A, OsCUL1, OsCUL3A and OsCUL4 were made individually in our laboratory. The presence of RBSDV in individual test samples was determined using the RBSDV P8 and P10 antibodies. The expression of OsCSN5A in rice plants was determined using the OsCSN5A‐specific antibody, and the accumulation of CUL/RUB‐CUL complexes in the tissue samples was determined using the OsCUL1‐, OsCUL3A‐ and OsCUL4‐specific antibodies. GFP‐specific antibody was purchased from TransGene (Beijing, China) and was used to detect OsJAZ8‐GFP expression in plant tissues.

### Yeast two‐hybrid (YTH) assay

The YTH assay was conducted using the Matchmaker Gold Yeast Two‐Hybrid System and the Yeastmaker Yeast Transformation System 2 (Clontech, Mountain View, CA, USA) as previously described (Yang *et al*., 2017).

### Co‐immunoprecipitation (Co‐IP) assay

Co‐IP assay was conducted as previously described (Mei *et al*., 2018), with modifications. *N. benthamiana* leaves were harvested, pooled and ground in liquid nitrogen. Leaf powders (0.5 g per sample) were transferred into individual 2 ml centrifuge tubes and mixed with 1 ml lysate buffer (50 mM Tris‐HCl, pH 7.5, supplemented with 150 mM NaCl, 10 mM MgCl_2_, 5 mM DTT, and 0.1% Triton X‐100). After 1 min vortexing and 20 min incubation on ice, the samples were centrifuged at 4°C for 10 min at 3000 ***g***. The resulting supernatants were transferred into individual spin columns containing 50 μl Sepharose bead, conjugated with an anti‐HA antibody, per column (Thermo Fisher Scientific, Waltham, MA, USA), incubated for 4 h at 4°C, and then centrifuged for 1 min at 3000 ***g***. Cold tris‐buffered saline (TBS) solution (50 mM Tris‐HCl, pH 7.5, 150 mM NaCl) was added to the columns followed by centrifugation at 4°C for 1 min at 3000 ***g***. After three washes with TBS, 30 μl elution buffer (5 mM ethylenediaminetetraacetic acid (EDTA) and 200 mM NH_4_OH) was added to each spin column and centrifuged for 20 s to elute the protein. The eluted proteins were collected in new tubes, boiled for 5 min, and then analyzed by Western blot analysis.

### Gel filtration analysis

Leaves were collected from the WT and P5‐1 transgenic rice plants and homogenized individually in the lysate buffer. Gel filtration analysis was performed as previously described (Giuliana *et al*., 2004).

### Agro‐infiltration of *N. benthamiana* leaves and confocal microscopy

Plasmids pGWB5C‐OsJAZ8 and pCV‐GFP were transformed individually into *Agrobacterium tumefaciens* strain GV3101 by electroporation. The *Agrobacterium* cultures were grown overnight, pelleted, re‐suspended in an induction buffer (1 M MgCl_2_ and 10 mM MES, pH 5.6, and 100 mM acetosyringone), and incubated for 3 h at room temperature before leaf infiltration. The infiltrated leaves were collected at 3 d post agro‐infiltration (dpai) and examined under a Leica TCS SP5 confocal laser scanning microscope (Leica Microsystems, Heidelberg, Germany).

### Hormone treatments

Methyl jasmonate (MeJA; Sigma‐Aldrich) was dissolved in a small amount of 100% ethanol (stock solution) and then diluted with sterile distilled water containing 0.1% Triton X‐100 to obtain a 50 μM solution immediately before use. Rice seedlings were sprayed with the 50 μM MeJA solution or with 0.1% Triton X‐100 as a control (mock). At 12 h post‐MeJA or ‐Triton X‐100 treatment, the seedlings were inoculated with RBSDV using viruliferous SBPHs. Each treatment had 45 rice seedlings, and RBSDV infection in these plants was determined by RT‐PCR using primers P1 and P2 (Table S1). For OsJAZ8‐GFP degradation assays, plasmids pGWB5C‐OsJAZ8‐GFP and pCV‐GFP were either agro‐infiltrated into *N. benthamiana* leaves or co‐infiltrated with pGWB511‐P5‐1. At *c*. 60 h post agro‐infiltration (hpai), the infiltrated leaves were sprayed with a 100 μM MeJA solution or a 0.1% Triton X‐100 solution (mock). Two hours later, the leaves were harvested and examined under the Leica TCS SP5 confocal laser scanning microscope. In addition, 50 μM MG132 was applied to plant leaves 12 h before observation.

### Analysis of JA in rice

Leaves were harvested from the assayed rice plants and used for JA extraction as described previously (Fu *et al*., 2012). Rice leaves from transgenic L10, li‐3 and t‐6 plants were collected, ground in liquid nitrogen and then mixed individually (200 mg leaf powder per sample) with 95 pmol ^2^H_5_‐JA. Next, 2ml methanol was added to each sample and mixed, and the mixture was incubated overnight at −20°C. After 15 min centrifugation at 25 000 ***g*** at 4°C, supernatant was collected from each sample and dried under nitrogen gas. The pellets were individually dissolved in 1 ml 5% ammonia solution and purified using the Oasis MAX SPE columns according to the manufacturer's instructions (Waters, Milford, MA, USA). The eluted JA was dried again under nitrogen gas, dissolved in 200 μl water : methanol mixture (20 : 80, v/v), and then analyzed by ultra‐high performance liquid chromatography/triple quadrupole mass spectrometry (UPLC‐MS/MS). Twenty independent biological replicates were analyzed for each treatment.

## Results

### P5‐1 facilitated RBSDV infection in rice

**Figure 1:**
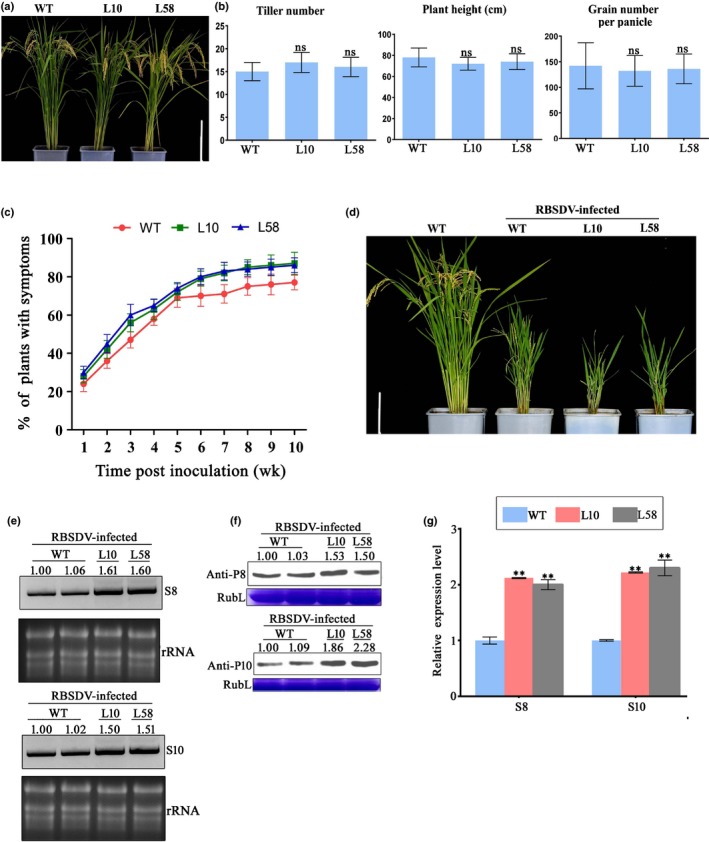
Transgenic *P5‐1* expression in rice (*Oryza sativa*) enhanced RBSDV infection. (a) Morphological comparisons between mature plants of the wild type (WT) and P5‐1 transgenic rice lines L10 and L58. Bar, 15 cm. (b) Quantitative measurements of tiller number, plant height, and grain number per panicle for the WT, L10 and L58 plants. Mean ± SD values are from three biological replicates with 15 plants per treatment, per replicate. ns, not significant according to Student's *t*‐test. (c) Time course study of RBSDV infection in the WT, L10 and L58 plants from the first to the 10^th^ week post virus inoculation. Inoculation assays were repeated three times. The error bars indicate SD. (d) Disease symptoms shown by RBSDV‐infected rice plants at 60 d post RBSDV inoculation. Plants labeled only ‘WT’ are healthy WT plants used as controls. Bar, 15 cm. (e) Northern blot analyses of RBSDV S8 and S10 RNA accumulations in the RBSDV‐inoculated WT, L10 and L58 plants. Ethidium bromide‐stained rRNA was used as a loading control. (f) Western blot analyses of RBSDV P8 and P10 protein accumulations in the RBSDV‐inoculated WT, L10 and L58 plants. Coomassie Blue‐stained Rubisco Large protein (RubL) was used as a loading control. The viral RNA and protein accumulation levels were quantified using imagej (US National Institutes of Health, http://rsb.info.nih.gov/nih-image/). (g) The relative RNA expression levels of RBSDV S8 and S10 in the RBSDV‐infected L10 and L58 plants vs the infected WT plants, from quantitative reverse transcription polymerase chain reaction (qRT‐PCR). (g) The relative expression levels of RBSDV S8 and S10, from qRT‐PCR. The expression level the of rice *actin* gene was used as an internal control. Mean ± SD values are from three biological replicates, each of which had three technical replicates. **, *P* < 0.01 according to Student's *t‐*test.

To establish the function of P5‐1 during RBSDV infection in rice, we generated two stable P5‐1 transgenic rice lines (L10 and L58). The expression level of *P5‐1* in these two lines was determined by RT‐PCR using *P5‐1* specific primers followed by Western blot analysis using a P5‐1 specific antibody (Fig. S1). The growth phenotypes of L10 and L58 plants were similar to that of the WT rice plants (Fig. 1a,b). After inoculation of L10, L58 and WT rice plants with RBSDV, the inoculated plants were grown inside a glasshouse for *c*. 10 wk, and RBSDV symptoms in the plants were recorded once per wk. The results showed that the percentages of L10 and L58 plants showing RBSDV symptoms were higher than that of the WT plants (Fig. 1c), and the L10 and L58 plants showed stronger stunting symptoms compared to the WT plants (Fig. 1d). In addition, the accumulation levels of RBSDV S8 and S10 RNAs as well as the P8 and P10 proteins were much higher in the infected L10 and L58 plants than in the infected WT plants (Fig. 1e–g), indicating that RBSDV P5‐1 protein can indeed facilitate RBSDV infection in rice.

### P5‐1 interacted with CSN subunit CSN5A

**Figure 2:**
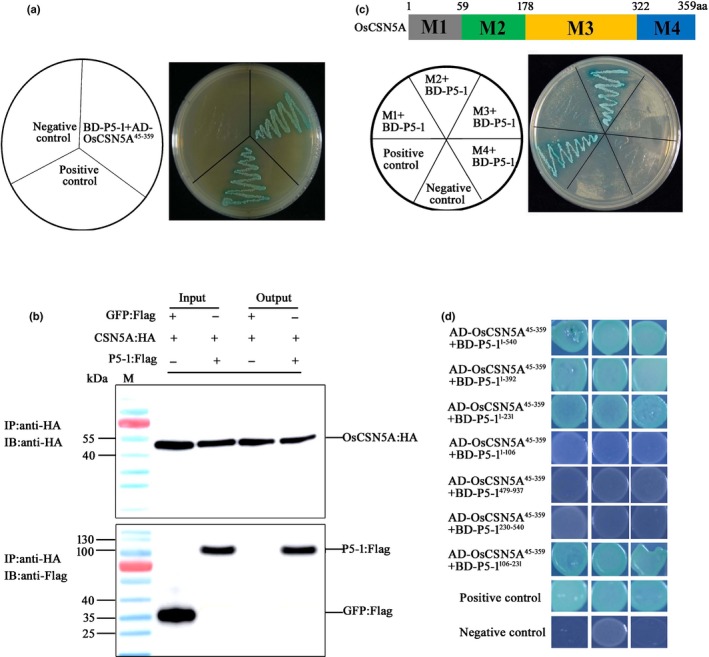
Interaction between RBSDV P5‐1 protein and rice (*Oryza sativa*) OsCSN5A. (a) RBSDV P5‐1 protein interacted with rice OsCSN5A in yeast cells. (b) Co‐immunoprecipitation assay showed that RBSDV P5‐1 interacted with rice OsCSN5A *in planta*. (c) RBSDV P5‐1 interacted with domain M2 (amino‐acid residues 59–178) in OsCSN5A. The colored strip shows the four domains in OsCSN5A, above which the amino acid positions of the four domains are indicated. (d) Determination of domain(s) in RBSDV P5‐1 that could interact with OsCSN5A. The results show that the domain covering amino‐acid residues 106–231 can interact with OsCSN5A.

To investigate how P5‐1 might enhance RBSDV infection in rice, we performed YTH assays using P5‐1 as bait to screen a rice cDNA library. From three independent screenings and *c*. 2 × 10^7^ cDNA clones, a total of 10 positive colonies were identified. Analysis of cDNA sequences representing these colonies revealed the presence of a polypeptide common to all 10 that contained 359 amino acids. Further sequence analysis showed that this polypeptide shared a sequence identity of 98.8% with the rice COP9 signalosome complex subunit 5a (OsCSN5A, XM_015781704), 77% with the Arabidopsis COP9 signalosome complex subunit 5A (AtCSN5A, AT1G22920) and 77% with the Arabidopsis COP9 signalosome complex subunit 5B (AtCSN5B, AT1G71230; Fig. S2). The identified gene was named *OsCSN5A*. Previous studies have shown that CSN5 can interact with the GAL4 DNA binding domain, but not with a truncated CSN5 lacking the N‐terminal 44 amino acids (Nordgard *et al*., 2001; Lozano‐Duran *et al*., 2011). These reports led us to construct a recombinant pAD‐OsCSN5A^45‐359^ plasmid and use it in a YTH assay. Our results showed that the BD‐P5‐1 interacted with AD‐OsCSN5A^45‐359^ in yeast cells (Fig. 2a). Interaction between P5‐1‐Flag and OsCSN5A‐HA *in planta* was then confirmed by a co‐immunoprecipitation (CO‐IP) assay after transient co‐expression of P5‐1‐Flag and OsCSN5A‐HA in *N. benthamiana* leaves (Fig. 2b). The results shown in Fig. 2(c) indicate that P5‐1 interacted with the M2 domain in OsCSN5A. More detailed analysis showed that a region covering amino acids 106–231 in P5‐1 played a crucial role in the interaction between P5‐1 and OsCSN5A (Fig. 2d).

### *OsCSN5A* expression in rice altered RBSDV infection but did not affect plant growth

**Figure 3:**
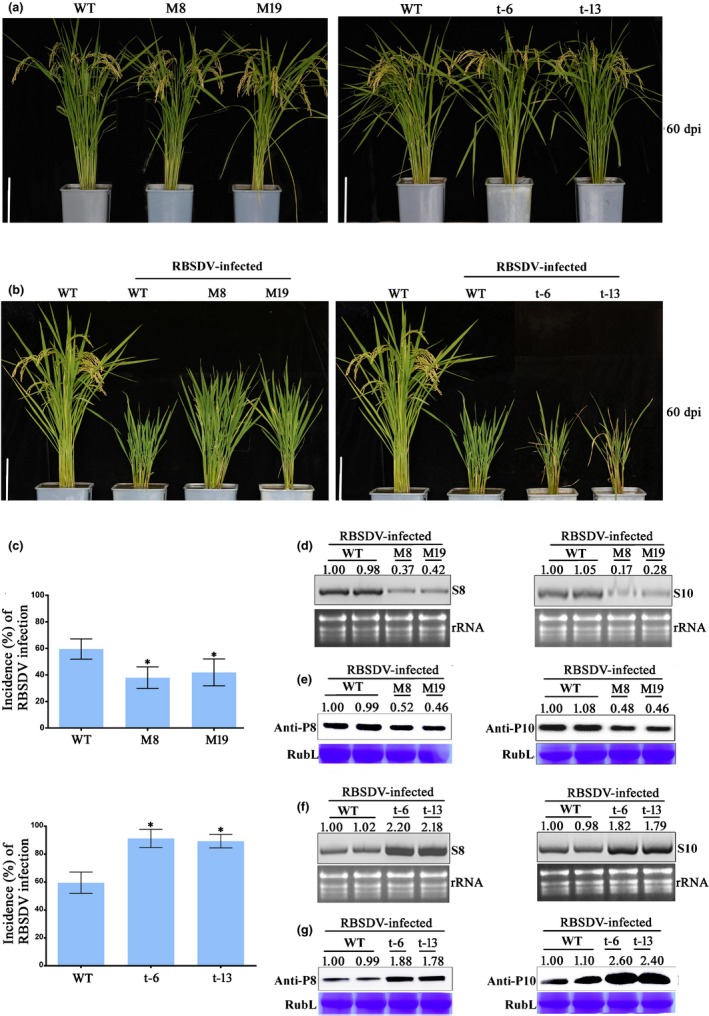
Alterations of *OsCSN5A* expression in rice (*Oryza sativa*) affected RBSDV infection but not plant growth. (a) Phenotypes of the wild‐type (WT), transgenic OsCSN5A rice line M8 and M19, and OsCSN5A‐silenced (RNAi) line t‐6 and t‐13 plants at maturity. Bars, 15 cm. (b) Phenotypes of the WT, line M8, line M19, line t‐6 and line t‐13 plants infected with RBSDV. The plants were photographed at 60 d post RBSDV inoculation. Bars, 15 cm. (c) Incidence of RBSDV infection in the WT, line M8 and line M19 plants, and in the WT, line t‐6 and line t‐13 plants. Mean ± SD values are from three independent experiments with 45 plants per experiment. *,* P *<* *0.05 according to Student's *t*‐test. (d) Northern blot analysis of RBSDV S8 and S10 RNA accumulations in the RBSDV‐infected WT, line M8 and line M19 plants. (e) Western blot analysis of RBSDV P8 and P10 protein accumulations in the RBSDV‐infected WT, and OsCSN5A OE line M8 and line M19 plants. (f) Northern blot analyses of RBSDV S8 and S10 RNsA accumulations in RBSDV‐infected WT, line t‐6 and line t‐13 plants. (g) Western blot analyses of RBSDV P8 and P10 protein accumulations in the RBSDV‐infected WT, line t‐6 and line t‐13 plants. Ethidium bromide‐stained rRNAs and the Coomassie Blue‐stained Rubisco Large protein (RubL) were used as RNA and protein loading controls, respectively. The viral RNA and protein accumulation levels were determined using imagej.

To determine whether OsCSN5A affects RBSDV infection in rice, we generated two stable transgenic rice lines (M8 and M19) with increased expression of OsCSN5A, and two OsCSN5A‐silenced (RNAi) transgenic rice lines (t‐6 and t‐13). Under glasshouse conditions, these transgenic plants all showed normal growth phenotypes (Fig. 3a). The results of qRT‐PCR confirmed that the expression level of *OsCSN5A* in the M8 and M19 plants was higher than that observed in the WT control plants, while the expression level of *OsCSN5A* in the t‐6 and t‐13 plants was lower than in the WT (Fig. S3). We then inoculated the M8, M19, t‐6 and t‐13 plants with RBSDV using RBSDV viruliferous SBPHs. The results showed that the RBSDV‐inoculated M8 and M19 plants showed milder RBSDV symptoms than the RBSDV‐inoculated WT control plants. By contrast, the RBSDV‐inoculated t‐6 and t‐13 plants showed stronger RBSDV symptoms than the RBSDV‐inoculated WT control plants at 60 dpi (Fig. 3b). Additionally, the incidence of RBSDV infection in the RBSDV‐inoculated M8 and M19 plants was significantly lower than in the RBSDV‐inoculated WT control plants, while it was significantly higher in the RBSDV‐inoculated t‐6 and t‐13 plants than in the RBSDV‐inoculated WT control plants (Fig. 3c). Northern blot analysis showed that the accumulations of RBSDV S8 and S10 RNAs in the RBSDV‐inoculated M8 and M19 plants were significantly reduced relative to those observed in the WT control plants (Fig. 3d). This finding was supported by the Western blot results, which showed that much lower accumulations of RBSDV P8 and P10 proteins were observed in the RBSDV‐inoculated M8 and M19 plants than in the WT control plants (Fig. 3e). By contrast, the accumulations of RBSDV S8 and S10 RNAs and proteins in the RBSDV‐inoculated t‐6 and t‐13 plants were significantly higher than those observed in the WT control plants (Fig. 3f–g). We therefore conclude that OsCSN5A is capable of regulating RBSDV replication and disease symptoms in infected rice plants.

### P5‐1 specifically interfered with CUL1 deRUBylation

**Figure 4:**
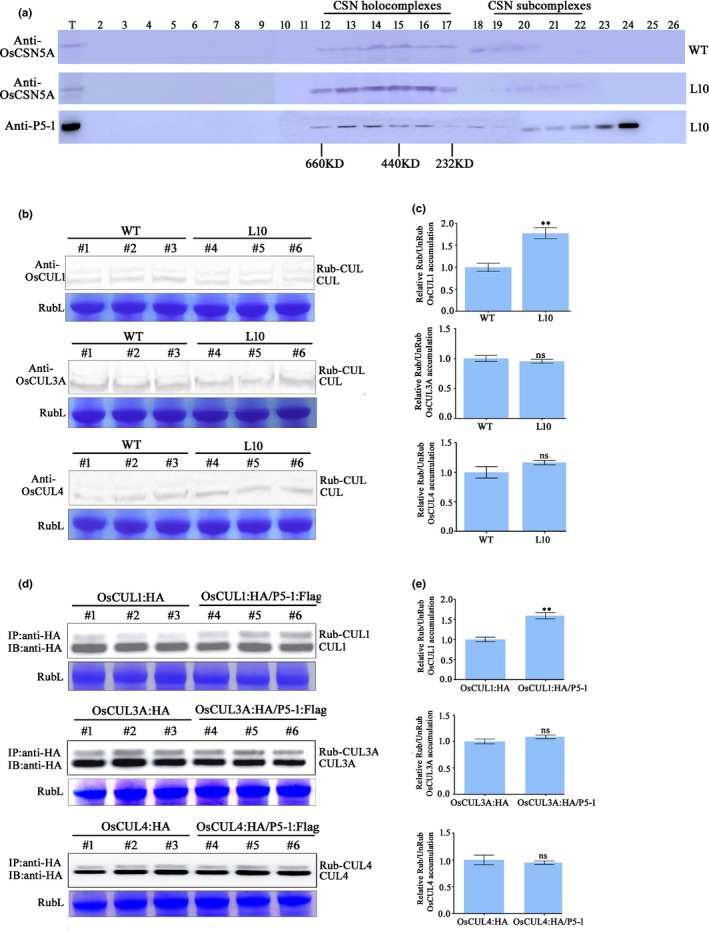
Western blot analyses of OsCSN5A accumulations in wild type (WT) and P5‐1 transgenic plants. (a) Tissues were harvested from 8‐wk‐old WT and L10 plants. Tissues from line L10 plants were pooled (referred to as L10) before use. After gel filtration, the samples were analyzed in SDS‐PAGE gels followed by detection using an OsCSN5A‐specific antibody. T, total unfractionated extract. (b) Western blot analyses of OsCUL1, OsCUL3a and OsCUL4 accumulations in the WT and L10 samples using OsCUL1‐ (top), OsCUL3a‐ (middle) and OsCUL4‐ (bottom) specific antibodies. Coomassie Blue‐stained Rubisco Large protein (RubL) was used as a loading control. The RUBylated (RUB‐CUL) and deRUBylated cullins (CUL) are indicated. (c) Ratios between the RUBylated and deRUBylated CUL1 (top), CUL3a (middle) and CUL4 (bottom) in the WT and the L10 samples. The protein accumulation levels were determined using imagej. The error bars indicate ± SD. **,* P *<* *0.01; ns, not significant according to Student's *t*‐test. (d) Western blot analyses of OsCUL1 (top), OsCUL3a (middle) and OsCUL4 (bottom) accumulations in *Nicotiana benthamiana* leaves using a HA antibody. Coomassie Blue‐stained Rubisco Large protein (RubL) was used as a loading control. RUBylated (Rub‐CUL) and deRUBylated cullins (CUL) are indicated. (e) Ratios of RUBylated : deRUBylated CUL1 (top), CUL3a (middle) and CUL4 (bottom) in *N. benthamiana* leaves transiently expressing OsCUL:HA and both OsCUL:HA and P5‐1:Flag are shown. The protein accumulation levels were determined using imagej. The error bars indicate ± SD. **,* P *<* *0.01; ns, not significant according to Student's *t*‐test.

Based on the above findings, we speculated that P5‐1 might sequester OsCSN5A to prevent the formation of COP9 complexes or may associate directly with the CSN holocomplexes through interaction with OsCSN5A. To test this hypothesis, we conducted a gel filtration experiment using extracts from WT and L10 plants using OsCSN5A and a P5‐1 specific antibody. Protein blot analyses indicated that the protein extracts from the WT plants were eluted in high molecular mass fractions (Fig. 4a), which may correspond to the CSN complex (fractions 12–17; Gusmaroli *et al*., 2007). A comparison between the gel filtration profiles of WT and L10 plants demonstrated that P5‐1 was indeed associated with both the CSN holocomplex (fractions 12–17), where it exercises its deRUBylation activity, and subcomplex(fractions 19–22)forms (Fig. 4a). We then tested whether the interaction between P5‐1 and OsCSN5A affects the deRUBylation activity of CSN complexes by comparing the relative levels of RUBylated and deRUBylated cullins in the WT rice plants with those in the L10 plants. Total protein was extracted from the WT and L10 plants, respectively, and then subjected to Western blot analyses using antibodies specific to OsCUL1, OsCUL3A and OsCUL4. The results showed that the relative levels of RUBylated OsCUL1 in the L10 plants was significantly higher than in the WT plants. The relative levels of RUBylated OsCUL3A and OsCUL4 were, however, not altered relative to that of the WT control plants (Fig. 4b,c). To obtain further support for these results, we individually transiently expressed OsCUL1‐HA, OsCUL3A‐HA and OsCUL4‐HA fusion protein alone or expressed together with P5‐1‐Flag in *N. benthamiana* leaves through agro‐infiltration. At 3 dpai, the samples were collected, immunoprecipitated with a HA antibody and subjected to Western blot analyses. The results showed that the relative levels of RUBylated OsCUL1 in the co‐expression plants were significantly higher than in the individual expression plants, whereas we did not observe clear changes in the relative levels of RUBylated OsCUL3A and OsCUL4 between the co‐expression plants and the individual expression plants (Fig. 4d,e). These findings suggest that P5‐1 specifically interferes with CSN‐mediated deRUBylation of OsCUL1.

### Silencing *OsCUL‐1* gene expression in rice enhanced RBSDV infection

**Figure 5:**
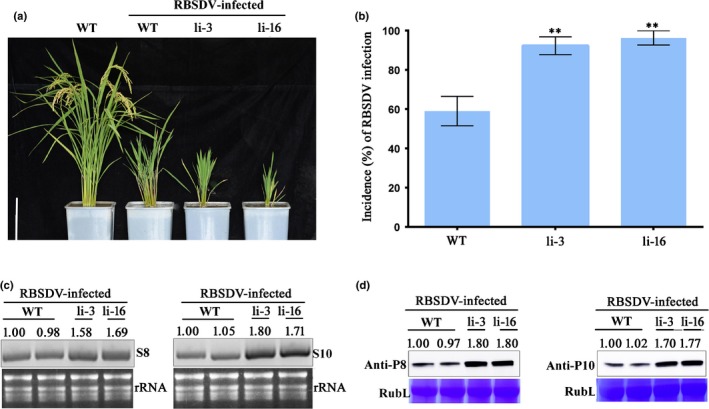
Silencing *OsCUL‐1* expression in rice (*Oryza sativa*) enhanced RBSDV infection. (a) RBSDV symptoms shown by the infected wild‐type (WT), OsCUL1 RNAi li‐3 and li‐16 plants at 60 d post RBSDV inoculation (dpi). Plants labeled only ‘WT’ are healthy WT plants used as controls. Bar, 15 cm. (b) RBSDV incidence in WT, li‐3 and li‐16 plants. Mean ± SD values are from three independent experiments with 45 plants per treatment. **,* P *<* *0.01 according to Student's *t*‐test. (c) Northern blot analysis of RBSDV S8 and S10 RNA accumulations in the RBSDV‐inoculated WT, li‐3 and li‐16 plants. Ethidium bromide‐stained rRNA was used as a loading control. (d) Western blot analysis of RBSDV P8 and P10 protein accumulations in the RBSDV‐inoculated WT, li‐3 and li‐16 plants. Coomassie Blue‐stained Rubisco Large protein (RubL) was used as a loading control. The viral RNA and protein accumulation levels were determined using imagej.

To investigate the function of OsCUL1 in RBSDV infection, we generated two OsCUL1‐silenced transgenic rice lines: li‐3 and li‐16. Under glasshouse conditions, most li‐3 and li‐16 plants showed normal growth, but some plants showed embryonic lethal phenotypes (data not shown), similar to the phenotype reported for the Arabidopsis CUL1 null mutant plants (Hellmann *et al*., 2003). Analyses by qRT‐PCR and Western blot showed that both RNA and protein expression levels of *OsCUL1* in the li‐3 and li‐16 plants were indeed significantly decreased (Fig. S4a,b). Inoculation of the WT, li‐3 and li‐16 plants using RBSDV viruliferous SBPHs showed that by 60 dpi, the RBSDV‐inoculated li‐3 and li‐16 plants showed stronger RBSDV symptoms than the RBSDV‐inoculated WT rice plants (Fig. 5a). In addition, the incidence of RBSDV infection observed in the RBSDV‐inoculated li‐3 and li‐16 plants was significantly increased (Fig. 5b). Northern blot analysis showed that the levels of RBSDV S8 and S10 RNAs in the RBSDV‐inoculated li‐3 and li‐16 plants were significantly higher than in the RBSDV‐inoculated WT plants (Fig. 5c). The results of Western blot analysis agreed those from the Northern blotting, and showed that the levels of RBSDV P8 and P10 proteins in the RBSDV‐inoculated li‐3 and li‐16 plants were much higher than those observed in the RBSDV‐inoculated WT plants (Fig. 5d).

### Jasmonic acid signaling was suppressed in transgenic P5‐1 rice

**Figure 6:**
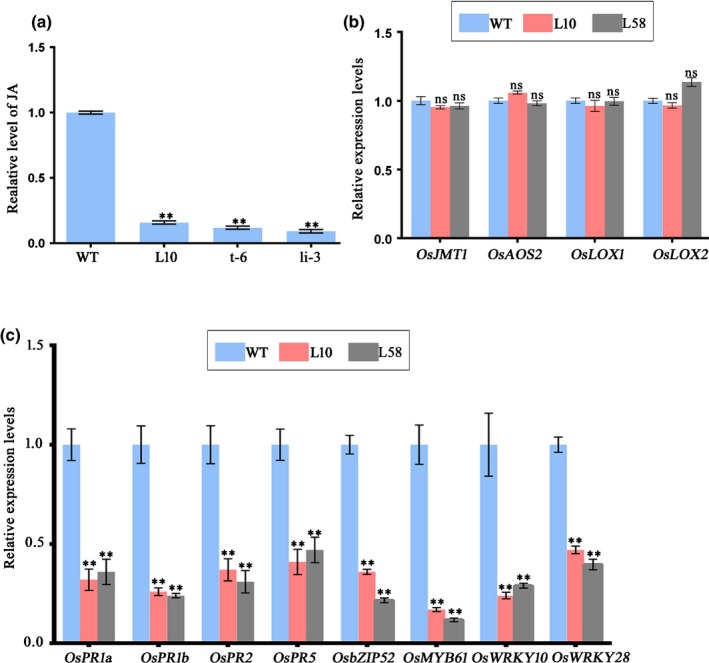
Effects of P5‐1, OsCSN5A and OsCUL1 on jasmonate (JA) production and gene expression. (a) Detection of endogenous JA production in wild‐type (WT), L10, t‐6 and li‐3 plants. Mean ± SD values are from three independent experiments with 20 plants per treatment, per experiment. (b) Quantitative reverse transcription polymerase chain reaction (qRT‐PCR) analysis of expressions of four JA biosynthesis‐related genes in the WT, L10 and L58 plants. Mean ± SD values are from three biological replicates, and each replicate had three technical replicates. ns, not significant according to Student's *t*‐test. (c) qRT‐PCR analysis of JA‐responsive genes in the WT, L10 and L58 plants. The expression level of the rice *actin* gene was used as an internal control. Mean ± SD values are from three biological replicates. **,* P *<* *0.01 according to Student's *t*‐test.

The SCF complex has been reported to be important in the regulation of plant hormone signaling pathways, and *cul1* mutant plants have been shown to exhibit altered JA responses (Moon *et al*., 2007; Thines *et al*., 2007; Gilkerson *et al*., 2009). In this study, we investigated the influence of P5‐1 on plant hormone signaling by comparing the levels of JA, ethylene (ET), gibberellic acid (GA_3_) and indole‐3‐acetic acid (IAA) in the L10 plants tissues with that in the WT plant tissues. The results indicated that the concentration of JA in the L10 tissues was significantly reduced compared to that in the WT plant tissues, and we did not observe clear changes in the concentrations of other plant hormones (Figs 6a, S5). Similarly, we also found that JA concentrations in the t‐6 and li‐3 plants’ tissues were also significantly reduced compared that that observed in the WT plants (Fig. 6a). To confirm this finding, we analyzed the expressions of JA‐responsive genes *OsPR1a*,* OsPR1b*,* OsPR2*,* OsPR5*,* OsbZIP52*,* OsMYB61*,* OsWRKY10* and *OsWRKY28* in the WT, L10 and L58 plants using qRT‐PCR. The results of this experiment showed that the expressions of these JA‐responsive genes were significantly down‐regulated in both transgenic plants compared to the WT plants (Fig. 6c). In a separate experiment, we analyzed the expressions of JA synthesis‐related genes *jasmonate O‐methyltransferase* (*OsJMT1*), *allene oxide synthase 2* (*OsAOS2*) and *lipoxygenases* (*OsLOXs*) through qRT‐PCR. We demonstrated that the expressions of these three JA synthesis‐related genes were not changed in the transgenic plants relative to the expression in the WT plants (Fig. 6b), implying that RBSDV‐encoded P5‐1 does not impair JA biosynthesis but does hinder JA responses.

### P5‐1 inhibited the degradation of OsJAZ8

**Figure 7:**
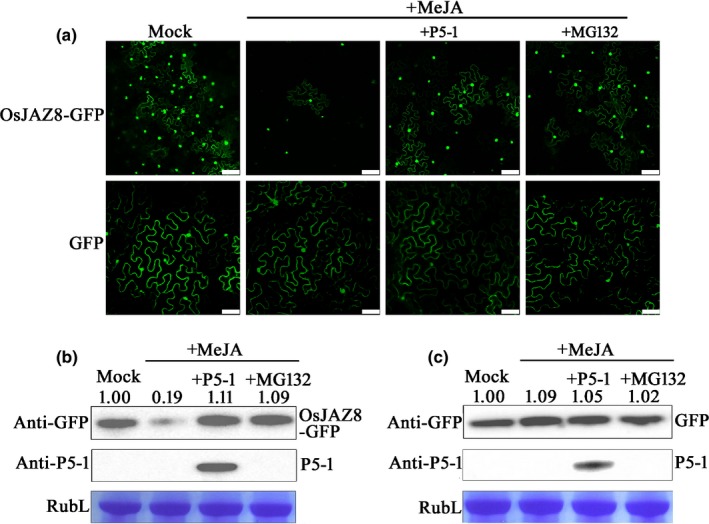
*In vivo* OsJAZ8‐GFP degradation assay. (a) A construct expressing OsJAZ8‐GFP or GFP only was agro‐infiltrated, or agro‐infiltrated together with a construct expressing P5‐1‐Flag, into *Nicotiana benthamiana* leaves. At 60 h post agro‐infiltration, the infiltrated leaves were sprayed with 100 μM methyl jasmonate (MeJA) or with 0.1% Triton X‐100 (mock). After 1–2h, the infiltrated leaves were harvested and examined under a confocal microscope. 50 μM MG132 was applied to plant leaves 12 h before observation. Bars, 100 μm. (b) Western blot analyses of OsJAZ8‐GFP and P5‐1 accumulations in various assayed leaves using a GFP and a P5‐1 specific antibody. (c) Western blot analyses of GFP and P5‐1 accumulations in various assayed leaves using a GFP‐specific and a P5‐1‐specific antibody. Coomassie Blue‐stained Rubisco Large (RubL) was used as a loading control.

Arabidopsis JASMONATE ZIM‐domain (JAZ) proteins are known to be transcriptional repressors of JA‐responsive genes (Yan *et al*., 2007). When JA concentrations are elevated in plant tissues, JAZ proteins are degraded by the 26S proteasome in an SCF^COI1^‐dependent manner, leading to rapid activations of JA‐responsive genes (Pauwels & Goossens, 2011). OsJAZ8 is a JAZ protein and can be degraded by SCF^COI1^ in the presence of JA (Yamada *et al*., 2012). To investigate whether P5‐1 could also regulate the function of the SCF complex and induce accumulations of SCF complex substrates, we transiently expressed OsJAZ8‐GFP fusion protein in *N. benthamiana* leaves through agro‐infiltration. By 72 h post agro‐infiltration, green fluorescence from the OsJAZ8‐GFP fusion protein was observed in the nuclei of the *N. benthamiana* leaf cells (Fig. 7a). As expected, after the leaves were treated with 100 μM MeJA, the green fluorescence was diminished. This finding indicates that the MeJA treatment triggered the SCF^COI1^‐mediated ubiquitination of OsJAZ8‐GFP fusion protein, followed by the degradation of this fusion protein by the 26S proteasome (Fig. 7a). By contrast, when P5‐1 and OsJAZ8‐GFP were co‐expressed in *N. benthamiana* leaves, the effect of MeJA treatment was significantly reduced. Similarly, when the OsJAZ8‐GFP expressing *N. benthamiana* leaves were treated simultaneously with MeJA and MG132, a specific inhibitor of the 26S proteasome, the effect of MeJA treatment was inhibited (Fig. 7a). This result was further supported by Western blot analysis, using an anti‐GFP antibody (Fig. 7b). In this experiment, *N. benthamiana* expressing GFP alone was used as a control. Co‐expression of P5‐1 and GFP in *N. benthamiana* leaves was observed following MeJA treatment, while treatment with both MeJA and MG132 resulted in GFP expression only, as observed in the mock‐treated leaves (Fig. 7a–c). These results demonstrate that P5‐1 is able to inhibit the SCF^COI1^‐dependent ubiquitination pathway.

### Jasmonate treatment did not affect RBSDV infection in P5‐1 transgenic plants

**Figure 8:**
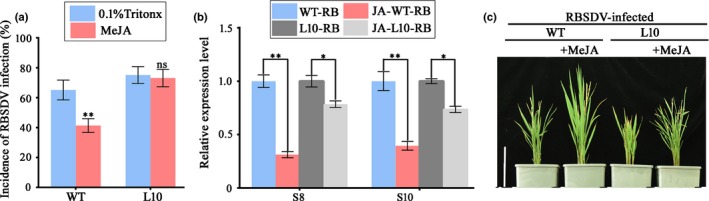
Effects of MeJA application on RBSDV infection in rice (*Oryza sativa*). (a) Wild type (WT) and L10 seedlings were sprayed with 50 μM MeJA or with 0.1% Triton X‐100 only (mock), followed by RBSDV inoculation using RBSDV viruliferous SBPHs. The disease incidences were determined at 60 d post RBSDV inoculation. Mean ± SD values are from three independent experiments with 45 plants per treatment, per experiment. **,* P *<* *0.01; ns, not significant according to Student's *t*‐test. (b) The relative expression levels of RBSDV S8 and S10 from qRT‐PCR. The expression level of the rice *actin* gene was used as internal control. Mean ± SD values are from three biological replicates, each of which had three technical replicates. *,* P *<* *0.05 **, *P* < 0.01, Student's *t‐*test. (c) The phenotype of WT and L10 seedlings sprayed with 50 μM MeJA or with 0.1% Triton X‐100 only (mock) followed by RBSDV inoculation using RBSDV viruliferous SBPHs at 60 d post RBSDV inoculation (dpi). Bar, 15 cm.

Because JA signaling plays a significant role in plant defense responses, we decided to investigate whether suppression of JA accumulation in the L10 plants could enhance RBSDV infection in rice. Seedlings of the WT and L10 plants were treated with 50 μM MeJA or with 0.1% Triton X‐100 (mock). At 12 h post‐treatment, the seedlings were inoculated with RBSDV viruliferous or non‐viruliferous SBPHs (three SBPHs per seedling and over 40 seedlings per treatment) for 3 d. The RBSDV‐infected plants were identified using RT‐PCR at 60 dpi. The results from this study showed that the incidence of RBSDV infection shown by the P5‐1 expressing plants was not affected by the MeJA treatment. By contrast, the MeJA treatment did reduce RBSDV incidence in the WT plants (Fig. 8a). In addition, the results of qRT‐PCR showed that the MeJA treatment significantly reduced the expression levels of S8 and S10 of RBSDV in WT plants compared to that observed in L10 plants (Fig. 8b). However, RBSDV symptoms in the WT plants as well as the L10 plants were alleviated after MeJA treatment (Fig. 8c).

## Discussion

Numerous studies have demonstrated that to acheive infection in plants, viruses have evolved to encode factor(s) for the defeat of plant defense machinery. For example, *Rice dwarf virus* (RDV)‐encoded P2 protein was shown to target OsIAA10 protein to ensure its infection in rice (Jin *et al*., 2014). *Cotton leaf curl Multan virus* (CLCuMuB)‐encoded βC1 protein was shown to interact with NbSKP1 to inhibit the ubiquitination activity of SCF E3 ligases, leading to a more severe CLCuMuV infection in *N. benthamiana* (Jia *et al*., 2016). *Tobacco mosaic virus* (TMV) replication‐associated protein was shown to disrupt the localization and stability of auxin/indole‐3‐acetic acid (IAA) proteins in Arabidopsis to promote its infection (Padmanabhan *et al*., 2008). To further establish the molecular mechanism(s) regulating plant–virus arms races, we conducted detailed analyses to understand how RBSDV regulates the cellular machinery of rice to benefit its infection. The results from this study showed that increased expression of RBSDV P5‐1 protein in transgenic rice plants did not affect rice growth but enhanced RBSDV RNA accumulations and disease symptom severity (Fig. 1). Through various assays, we determined that RBSDV P5‐1 protein interacted specifically with OsCSN5A, an important component in the COP signalosome (CSN) complexes (Fig. 2). We also demonstrated that increased expression of OsCSN5A in transgenic rice plants reduced the incidence of RBSDV infection. This finding was supported by the fact that RBSDV infection in rice can be promoted through knockdown of *OsCSN5A* in rice via stable transformation (Fig. 3). Liu *et al*. (2002) reported previously that silencing COP9 signalosome expression in *N. tabacum* compromised the *N* gene‐mediated host resistance to TMV infection. Our findings demonstrate that RBSDV P5‐1 protein can bind directly to OsCSN5A in rice cells to enhance RBSDV accumulation, leading to more severe disease symptoms.

CSN5 is known to contain an Mpr1/Pad1 N‐terminal (MPN) domain that is crucial for the removal of RUB modification from the cullin subunit in the CRL ubiquitin ligase complexes (Lyapina *et al*., 2001; Cope *et al*., 2002). In this study, we found that a region located within the OsCSN5A MPN domain may interact with P5‐1 (Fig. 2). An earlier report by Gusmaroli *et al*. (2007) showed that depletion of the MPN domain from CSN5A or CSN5B resulted in an inactivation of CSN and a loss of deRUBylation by CUL1, CUL3 and CUL4 in Arabidopsis. We speculated that the loss of OsCSN5A isopeptidase activity was likely caused by the interaction between P5‐1 and OsCSN5A. To test this idea, we analyzed the ratio between RUBylated and deRUBylated CUL1, CUL3A and CUL4 in the L10 transgenic rice. The specificity of OsCUL1, OsCUL3A and OsCUL4 antibodies were analyzed by Western blot analysis (Fig. S6). The results showed that more RUBylated CUL1 had accumulated in the transgenic rice lines than deRUBylated CUL1. By contrast, the RUBylated : deRUBylated accumulation ratio was unchanged for CUL3A and CUL4 (Fig. 4b). An earlier report showed that over‐expression of geminivirus‐encoded C2 protein in Arabidopsis compromised the CSN‐mediated deRUBylation of CUL1 (Lozano‐Duran *et al*., 2011). In addition, several other studies have demonstrated that the CSN complex contains a CSN5 subunit, and the activity center in this subunit is able to regulate the activity of CRLs (Gusmaroli *et al*., 2007; Schwechheimer & Isono, 2010). Although interactions between viral factors and CRL components have been reported previously for DNA and RNA viruses (Zhang *et al*., 2001; Oh *et al*., 2006; Lozano‐Duran *et al*., 2011; Fu *et al*., 2012), it is not clear whether CSN complexes can interact with RBSDV‐encoded factor(s). A previous report by Tao *et al*. (2017) showed that the RBSDV P7‐2 protein functioned as an SCF complex component in rice. The authors suggested that the gibberellin signaling pathway might play a role in RBSDV infection in rice . Other studies have shown that viruses often hijack host CRLs to promote their infections in plants (Adeyemi *et al*., 2014; Collins & Kathleen, 2014). We therefore propose that suppression of CUL1 activity by P5‐1 during RBSDV infection in rice can benefit RBSDV pathogenicity. To support our hypothesis, we silenced *CUL1* expression in rice through stable transformation and carried out RBSDV inoculation using RBSDV viruliferous SBPHs. As expected, the CUL1‐silenced rice plants showed higher susceptibility to RBSDV infection when compared with the WT rice plants (Fig. 5). Although we have demonstrated the importance of the interaction between P5‐1 and CSN5 in RBSDV infection in rice, further investigation is required to understand the role of the ubiquitination pathway in RBSDV infection.

**Figure 9:**
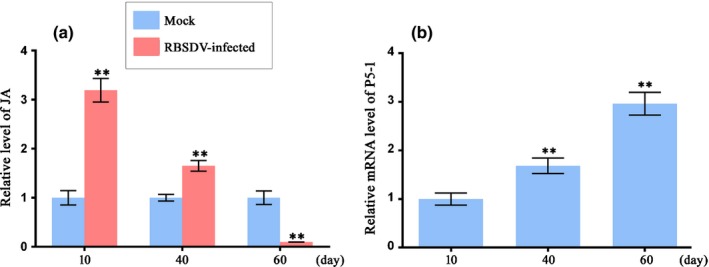
Effects of RBSDV infection on jasmonate (JA) production in rice (*Oryza sativa*). (a) Detection of endogenous JA production in the mock‐inoculated and RBSDV‐inoculated wild type rice plants. A total of 20 independent biological replicates were analyzed for each treatment. Mean ± SD values are from three independent experiments with 20 plants per treatment, per experiment. **, *P* < 0.01 according to Student's *t‐*test. (b) qRT‐PCR analyses of RBSDV P5‐1 RNA accumulation in the RBSDV‐infected plants at 10, 40 and 60 d post RBSDV inoculation (dpi). The expression level of the rice *actin* gene was used as an internal control. Mean ± SD values are from three independent experiments with three plants per treatment per experiment.

JA is an important plant signaling molecule that mediates plant biotic and abiotic stress responses. It also functions as a regulator of plant growth and development. It is noteworthy that a recent study reported an increase in JA production during the early stages of RBSDV infection in rice (He *et al*., 2017). In this study, we also observed that the production of JA in rice increased significantly upon RBSDV infection. As the disease progressed, JA concentrations in the infected plants declined continuously, reaching a level that was significantly lower than that observed in the uninfected control plants at 60 dpi (Fig. 9a). This result suggests that RBSDV has the ability to suppress JA biosynthesis in infected rice cells. This observation encouraged us to analyze the JA concentrations in L10 plants. Much lower concentrations of JA were detected in P5‐1 transgenic rice plants, indicating that P5‐1 may be related to the JA signal pathway (Fig. 6a). Indeed, we also found that JA production was inversely associated with the mRNA expression level of P5‐1 in RBSDV‐infected plants from 10 to 60 dpi (Fig. 9b). Previous studies have reported that preventing COI1 from being assembled into the SCF complexes in the *cul1* and *csn* mutant plants significantly reduces the production of JA (Feng *et al*., 2003; Ren *et al*., 2005). Our results, along with the findings of these studies, lead us to propose that RBSDV P5‐1 is a negative regulator of JA signaling during RBSDV infection and that its function is dosage dependent. The results presented here also show that expression of RBSDV P5‐1 in rice compromised the integrity of SCF^COI1^ (Fig. 7). A study by Sheard *et al*. (2010) demonstrated that after MeJA treatment, the JAZ proteins in the SCF^COI1^ complexes were degraded in a proteasome‐dependent manner . To determine whether P5‐1 could also interfere with JA signaling through the destabilization of SCF^COI1^ complexes, we analyzed the expressions of JA biosynthesis‐related genes in the L10 and L58 transgenic plants using qRT‐PCR. We found that over‐expression of P5‐1 in rice did not affect the expression of JA biosynthesis‐related genes (Fig. 6c) but did significantly suppress the expression of JA responsive‐genes (Fig. 6b). In this study, the WT rice plants treated with JA attenuated RBSDV infection, while application of MeJA to the L10 transgenic plants did not reduce their susceptibility to RBSDV infection (Fig. 8). This finding is in agreement with that of a previous report showing that MeJA treatment attenuated RBSDV infection in rice (He *et al*., 2017). *Tomato yellow leaf curl Sardinia virus*‐encoded C2 protein has also been shown to inhibit JA signaling through interaction with CSN5 (Lozano‐Duran *et al*., 2011). Several viral factors have been shown to interfere with host–vector interaction by inhibiting JA‐mediated signaling, which promotes retention of the vector on virus‐infected plants, thereby enhancing virus acquisition through prolonged phloem feeding (Zarate *et al*., 2007; Li *et al*., 2014; Carr *et al*., 2018). These findings are consistent with our observation that P5‐1 suppresses JA signaling to favour transmission by SBPH, though further experimental evidence is required to verify this result. In light of the evidence presented thus far, we speculate that P5‐1 is capable of interfering with host JA signaling to enhance RBSDV infection, as well as symptom development, in rice.

**Figure 10:**
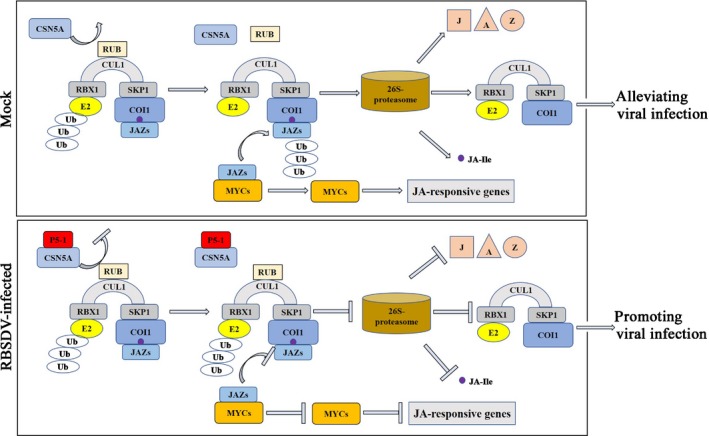
Proposed model. In mock rice plants (*Oryza sativa*), OsCSN5A removes the RUB1 from OsCUL1, regulating the activity of the SCF complex, leading to the ubiquitination and 26S proteasome degradation of OsJAZs, releasing the specific OsMYCs. Genes of the JA signaling pathway are then adequately regulated by corresponding MYC transcription factors, promoting normal rice growth. Under RBSDV infection condition, P5‐1 binds to OsCSN5A, compromising the activity of OsCSN5A over OsCUL1, and blocking OsJAZ degradation via ubiquitination and the 26S proteasome. The stabilized OsJAZs bind to corresponding MYCs, inhibiting down‐stream gene expression in the JA response signaling pathway and promoting RBSDV infection.

In summary, we have identified a novel strategy for controlling RBSDV infection in rice. Our results showed that the activity of the SCF complex is regulated through conjugation and removal of RUB1 from CULs via the metalloisopeptidase activity of CSN subunit CSN5. In healthy rice plants, COI1 of SCF^COI1^ functioned as a JA receptor through binding to JA conjugate JA‐isoleucine (JA‐Ile). The SCF^COI1^–JA‐Ile complex interacted with the JAZ repressor proteins, resulting in their polyubiquitination and subsequent proteasomal degradation. This derepression liberated an MYC transcription factor that is able to regulate the expressions of a subset of JA‐responsive genes, leading to attenuation of the RBSDV infection (Fig. 10a). Accumulation of P5‐1 during RBSDV infection in rice compromised the activity of CSN5A on CUL1. Accumulation of RUBylated CUL1 in cells prevented the proteasomal degradation of JAZs. JAZs interacted with MYCs to repress the expressions of a subset of JA‐responsive genes, resulting in the promotion of RBSDV infection in rice (Fig. 10b). Further studies are required to improve our understanding of the molecular mechanisms underlying this strategy and to provide new management strategies for RBSDV.

## Author contributions

HZ, JianY and JC conceived the project and designed the experiments; LH, JianY and HZ carried out the experiments with assistance from XC, JinY, TZ, JL, SZ and KZ; all authors analyzed and discussed the results; and JianY, HZ and JC wrote the manuscript.

## Supporting information

Please note: Wiley Blackwell are not responsible for the content or functionality of any Supporting Information supplied by the authors. Any queries (other than missing material) should be directed to the *New Phytologist* Central Office.

**Fig. S1** *P5‐1* expression levels detected in transgenic rice lines.
**Fig. S2** Multiple sequence alignment results.
**Fig. S3** Analysis of *OsCSN5A* expressions in the OsCSN5A over‐expression and silenced (RNAi) transgenic lines.
**Fig. S4** Analysis of *OsCUL1* expression in the wild‐type (WT) and *OsCUL1* gene silenced (RNAi) lines.
**Fig. S5** Relative levels of hormones in transgenic plants.
**Fig. S6** Specificity analysis of OsCUL1, OsCUL3A and OsCUL4 antibodies.

**Table S1** A list of primers used for vector constructions, quantitative PCR (qPCR), reverse transcriptase PCR (RT‐PCR), and preparation of Northern blot probes.
