# Supplementary material for: Rice black‐streaked dwarf virus‐encoded P5‐1 regulates the ubiquitination activity of SCF E3 ligases and inhibits jasmonate signaling to benefit its infection in rice
Source: New Phytol. 2019 Aug 9;225(2):896–912. doi: 10.1111/nph.16066 (PMC6972624; doi:10.1111/nph.16066)
Supplement: Supplementary file 1 — Fig. S1 P5‐1 expression levels detected in transgenic rice lines. Fig. S2 Multiple sequence alignment results. Fig. S3 Analysis of OsCSN5A expressions in the OsCSN5A over‐expression and silenced (RNAi) transgenic lines. Fig. S4 Analysis of OsCUL1 expression in the wild‐type (WT) and OsCUL1 gene silenced (RNAi) lines. Fig. S5 Relative levels of hormones in transgenic plants. Fig. S6 Specificity analysis of OsCUL1, OsCUL3A and OsCUL4 antibodies. [file NPH-225-896-s001.pdf]

New Phytologist Supporting Information

Article title: *Rice Black-Streaked Dwarf Virus* encoded P5-1 Regulates the Ubiquitination Activity of SCF E3 Ligases and Inhibits Jasmonate Signaling to Benefit Its Infection in Rice

Authors: Long He, Xuan Chen, Jin Yang, Tianye Zhang, Juan Li, Songbai Zhang, Kaili Zhong, Hengmu Zhang, Jianping Chen, Jian Yang

Article acceptance date: 09 July 2019

The following Supporting Information is available for this article:

Fig. S1 *P5-1* expression level was detected in transgenic rice lines.

Fig. S2 Multiple sequence alignment result.

Fig. S3 Analyses of *OsCSN5A* expressions in the OsCSN5A over-expression or silenced (RNAi) transgenic lines.

Fig.S4 Analyses of *OsCUL1* expression in the wild type (WT) or *OsCUL1* gene silenced (RNAi) lines.

Fig.S5 Relative levels of Hormones in Transgenic Plants.

Fig.S6 The specificity analyses of OsCUL1, OsCUL3A and OsCUL4 antibodies.

Table S1 A list of primers used for vector constructions, qPCR, RT-PCR, and preparation of Northern blot probes (separate Excel file)

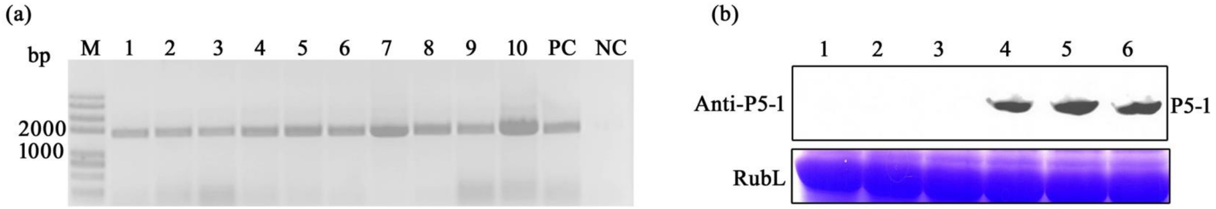

Fig. S1 *P5-1* expression level was detected in transgenic rice lines. (a), RT-PCR detection of *P5-1* expression in the T2 generation lines. NC, total RNA was isolated from a wild type (WT) rice plant and used as a negative control. PC, total RNA was isolated from a RBSDV-infected rice plant and used as a positive control. Lane 1 to Lane 10, total RNA was isolated from 10 different T2 transgenic lines. (b), Western blot assay using an antibody specific for RBSDV P5-1 protein. Lane 1 to 3, samples were prepared from the WT plants. Lane 4 to 6, samples were prepared from the T2 transgenic plants.

```
Polypeptide     MEPTSSAAMARQTWELENNIPAAASDPDALDAIYRYDEAAQARVQQEKPWANDPHPFRRA
OsCSN5A         MEPTSSAAMARQTWELENNIPAAASDPDALDAIYRYDEAAQARVQQEKPWANDPHPFRRA
AtCSN5A         ME-GSSSAIARKTWELENNILPVEPTDSASDSIFHYDDASQAKIQQEKPWASDPNYFKRV
AtCSN5B         ME-GSSSTIARKTWELENSILTVDSPDSTSDNIFYYDDTSQTRFQQEKPWENDPHYFKRV
                **  **...**.****** *        . * *. **...*.. ******  **. *.*

Polypeptide     KISALALLKMVVHARAGGTIEVMGLMQGKCEGDAIVVMDAFALPVEGTETRVNAQADAYE
OsCSN5A         KISALALLKMVVHARAGGTIEVMGLMQGKCEGDAIVVMDAFALPVEGTETRVNAQADAYE
AtCSN5A         HISALALLKMVVHARSGGTIEIMGLMQGKTEGDTIIVMDAFALPVEGTETRVNAQSDAYE
AtCSN5B         KISALALLKMVVHARSGGTIEIMGLMQGKTDGDTIIVMDAFALPVEGTETRVNAQDDAYE
                .**************.*****.*******..**.*.******************* ****

Polypeptide     YMVEYSTINKQAGRLENVVGWYHSHPGYGCWLSGIDVSTQMLNQQFQEPFLAVVIDPTRT
OsCSN5A         YMVEYSTINKQAGRLENVVGWYHSHPGYGCWLSGIDVSTQMLNQQFQEPFLAVVIDPTRT
AtCSN5A         YMVEYSQTSKLAGRLENVVGWYHSHPGYGCWLSGIDVSTQMLNQQYQEPFLAVVIDPTRT
AtCSN5B         YMVEYSQTNKLAGRLENVVGWYHSHPGYGCWLSGIDVSTQRLNQQHQEPFLAVVIDPTRT
                ******   * ***************************** **** **************

Polypeptide     VSAGKVEIGAFRTYPKDYKPPDEPVSEYQTIPLNKIEDFGVHCKQYYALDITYFKSSLDS
OsCSN5A         VSAGKVEIGAFRTYPKDYKPPDEPVSEYQTIPLNKIEDFGVHCKQYYALDITYFKSSLDS
AtCSN5A         VSAGKVEIGAFRTYPEGHKISDDHVSEYQTIPLNKIEDFGVHCKQYYSLDITYFKSSLDS
AtCSN5B         VSAGKVEIGAFRTYSKGYKPPDEPVSEYQTIPLNKIEDFGVHCKQYYSLDVTYFKSSLDS
                **************    * *. ***********************.**.*********

Polypeptide     HLLDLLWNKYWVNTLSSSPLLGNRDYVAGQIFDLADKLEQAEGQLAHSRYGMLMPS-QRK
OsCSN5A         HLLDLLWNKYWVNTLSSSPLLGNRDYVAGQIFDLADKLEQAEGQLAHSRYGMLMPS-QRK
AtCSN5A         HLLDLLGNKYWVNTLSSSPLLGNGDYVAGQISDLAEKLEQAESQLANSRYGGIAPAGHQR
AtCSN5B         HLLDLLWNKYWVNTLSSSPLLGNGDYVAGQISDLAEKLEQAESHLVQSRFGGVVPSSLHK
                ****** **************** ******* ***.****** .*  **.* . *.  ..

Polypeptide     KEQEESPLAKVTRDSSKITAEQVHGLMSQVIKDILFNSVHPSNKASTSAPDSSGPEPMVE
OsCSN5A         KEQEESPLAKVTRDSSKITAEQVHGLMSQVIKDILFNSVHPSNKASTSAPDSSGPEPMVE
AtCSN5A         RKEDEPQLAKITRDSAKITVEQVHGLMSQVIKDILFNSARQSK---KSADDSSDPEPMIT
AtCSN5B         KKEDESQLTKITRDSAKITVEQVHGLMSQVIKDELFNSMRQSNN--KSPTDSSDPDPMIT
                . ..*  *.*.****.*** ************* **** . *     *  *** *.**.
```

Fig. S2 Multiple sequence alignment result. Amino acid sequence of the identified polypeptide was aligned with the sequences of rice and *Arabidopsis* CSN5 sequences using the DNAMAN software. *, conserved amino acids.

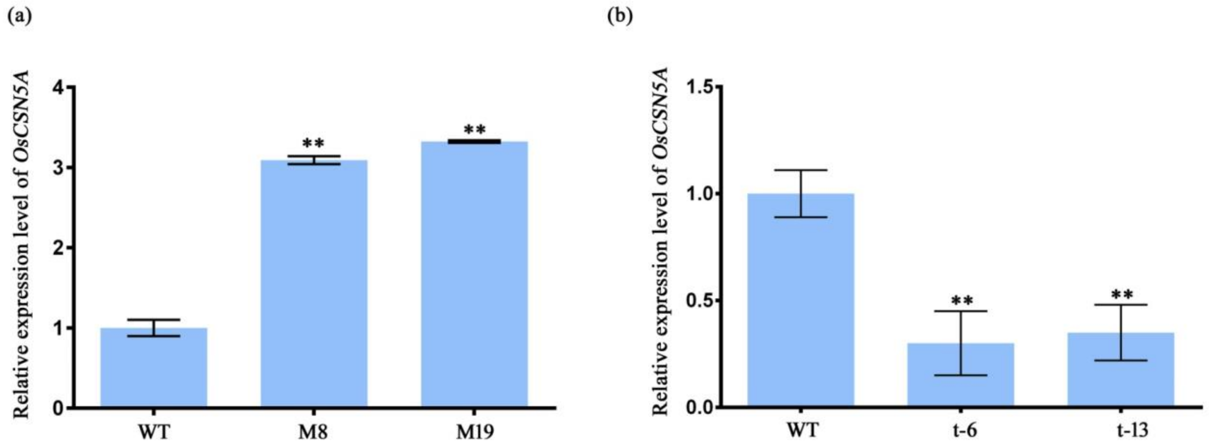

Fig. S3 Analyses of *OsCSN5A* expressions in the OsCSN5A over-expression or silenced (RNAi) transgenic lines. (a), qRT-PCR was used to determine the relative expression level of *OsCSN5A* in the T2 OsCSN5A over-expression M8 and M19 line plants. (b), qRT-PCR was used to determine the relative expression level of *OsCSN5A* in the T2 OsCSN5A RNAi t-6 and t-13 line plants. The expression level of rice *Actin* gene was used as an internal control. Each mean ± SD was from three biological replicates and each biological replicate had three technical replicates. **, $P < 0.01$ and was determined by the Student's *t*-test.

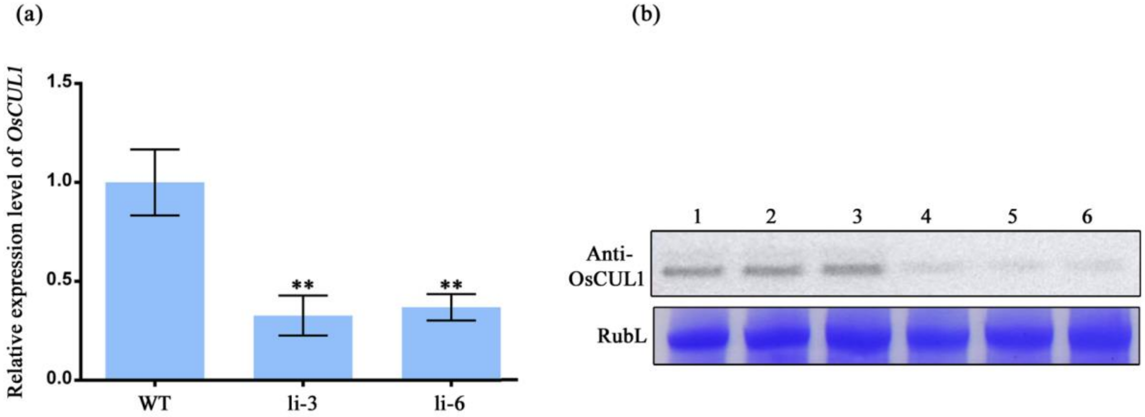

Fig. S4 Analyses of *OsCUL1* expression in the wild type (WT) or *OsCUL1* gene silenced (RNAi) lines. (a)**,** qRT-PCR was used to determine the relative expression of *OsCUL1* in the WT or the T2 OsCUL1 RNAi li-3 and li-16 line plants. The expression level of rice *Actin* gene was used as an internal control. Each mean ± SD was from three biological replicates and each biological replicate had three technical replicates. ****, *P*< 0.01 and was determined by the Student's *t*-test. (b), Western blot assay using an antibody specific for OsCUL1 protein. Lane 1 to 3, samples were prepared from the WT plants. Lane 4 to 6, samples were prepared from the T2 OsCUL1 RNAi li-3.

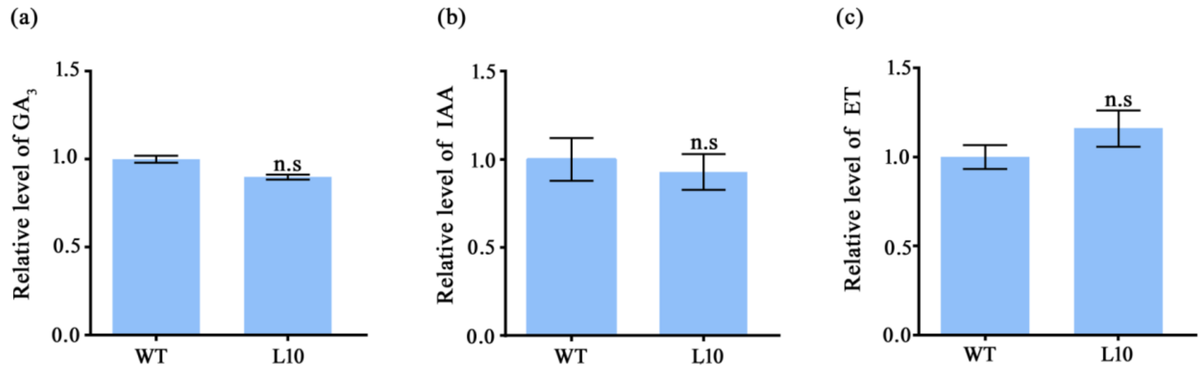

Fig. S5 Relative levels of Hormones in Transgenic Plants. (a), Relative level of GA3 in the WT and L10. Each mean ± SD was from three independent experiments with 20 plants per treatment per experiment. n.s., no significant difference based on the Student's *t*-test. (b), Relative level of IAA in the WT and L10. Each mean ± SD was from three independent experiments with 20 plants per treatment per experiment. n.s., no significant difference based on the Student's *t*-test. (c), Relative level of ET in the WT and L10. Each mean ± SD was from three independent experiments with 20 plants per treatment per experiment. n.s., no significant difference based on the Student's *t*-test.

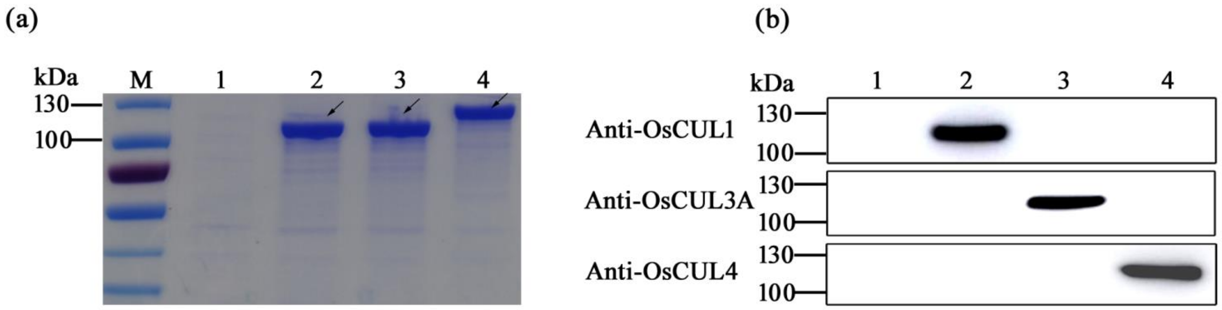

Fig. S6 The specificity analyses of OsCUL1, OsCUL3A and OsCUL4 antibodies. (a), SDS-PAGE analysis of the lysates of pET32a (Lane1), pET32a-OsCUL1(Lane2), pET32a-OsCUL3A (Lane3) and pET32a-OsCUL4 (Lane4) transformed BL21 playS induced by IPTG for 3 hours. (b), Western blot analyses of lysates of pET32a (Lane1), pET32a-OsCUL1 (Lane2), pET32a-OsCUL3A (Lane3) and pET32a-OsCUL4 (Lane4) transformed BL21 playS induced by IPTG for 3 hours.
